# Supplementary material for: In-Host HEV Quasispecies Evolution Shows the Limits of Mutagenic Antiviral Treatments
Source: Int J Mol Sci. 2023 Dec 6;24(24):17185. doi: 10.3390/ijms242417185 (PMC10743355; doi:10.3390/ijms242417185)
Supplement: Supplementary file 1 [file ijms-24-17185-s001.zip › ijms-2740500-supplementary.pdf]

Article

In-Host HEV Quasispecies Evolution Shows the Limits of Mutagenic Antiviral Treatments

Sergi Colomer-Castell [1,2,3,†], Josep Gregori [1,*,†], Damir Garcia-Cehic [1,2], Mar Riveiro-Barciela [1,2,4], Maria Buti [1,2,4], Ariadna Rando-Segura [2,5], Judit Vico-Romero [1], Carolina Campos [1,2,3], Marta Ibañez-Lligoña [1,4], Caroline Melanie Adombi [1,6], Maria Francesca Cortese [2,7], David Tabernero [2,7], Juan Ignacio Esteban [1,2,4], Francisco Rodriguez-Frias [2,7] and Josep Quer [1,2,3,*]

1 Liver Diseases-Viral Hepatitis, Liver Unit, Vall d'Hebron Institut de Recerca (VHIR), Vall d'Hebron Hospital Universitari, Vall d'Hebron Barcelona Hospital Campus, Passeig Vall d'Hebron 119-129, 08035 Barcelona, Spain; sergi.colomer@vhir.org (S.C.-C.); damir.garcia@vhir.org (D.G.-C.); mar.riveiro@gmail.com (M.R.-B.); maria.buti@vallhebron.cat (M.B.); judit.vico@vhir.org (J.V.-R.); carolina.campos@vhir.org (C.C.); marta.ibanez@vhir.org (M.I.-L.); adombicaro@yahoo.fr (C.M.A.); juanignacio.esteban@vallhebron.cat (J.I.E.)

2 Centro de Investigación Biomédica en Red de Enfermedades Hepáticas y Digestivas (CIBERehd), Instituto de Salud Carlos III, Av. Monforte de Lemos, 3-5, 28029 Madrid, Spain; ariadna.rando@vallhebron.cat (A.R.-S.); maria.cortese@vhir.org (M.F.C.); dtaberc@gmail.com (D.T.); frarodri@gmail.com (F.R.-F.)

3 Biochemistry and Molecular Biology Department, Universitat Autònoma de Barcelona (UAB), Campus de la UAB, Plaça Cívica, 08193 Bellaterra, Spain

4 Medicine Department, Universitat Autònoma de Barcelona (UAB), Campus de la UAB, Plaça Cívica, 08193 Bellaterra, Spain

5 Microbiology Department, Vall d'Hebron Institut de Recerca (VHIR), Vall d'Hebron Hospital Universitari, Vall d'Hebron Barcelona Hospital Campus, Passeig Vall d'Hebron 119-129, 08035 Barcelona, Spain

6 Institute of Agropastoral Management, University Peleforo Gon Coulibaly, Korhogo BP 1328, Côte d'Ivoire

7 Biochemistry Department, Vall d'Hebron Institut de Recerca (VHIR), Vall d'Hebron Hospital Universitari, Vall d'Hebron Barcelona Hospital Campus, Passeig Vall d'Hebron 119-129, 08035 Barcelona, Spain

* Correspondence: josep.gregori@gmail.com or josep.gregori@vhir.org (J.G.); josep.quer@vhir.org (J.Q.)

† These authors contributed equally to this work.

SUPPLEMENTARY METHODS

Sample size dependence. Rarefaction.

Diversity indices are dependent to a varying extent on the sample size[1], and this dependence has to be considered when comparing values from different samples. We used rarefaction to correct differences due to sample size. Briefly, from the set of samples to be compared, the minimum coverage (147,463 reads) was taken as the sample size reference. Each sample then underwent 1000 resample cycles, taking the frequencies of all haplotypes in the sample as probabilities, and the reference size as the resampling size. In each cycle, each diversity index was calculated from the resulting sampling. At the end of the 1000 cycles, averages and standard deviations were computed for each diversity index. In this way all diversity index values were normalized to the same sample size (coverage).

Quasispecies fitness partition

At a given time, a quasispecies is usually comprised of a highly predominant (master) haplotype, a few low- to medium-frequency genomes, various rare haplotypes with very low fitness but still able to replicate to some level, and some defective genomes unable to replicate[2]. Within the characteristic dynamics of a quasispecies, the fitness of a haplotype may be approximated by its frequency in the quasispecies. As a consequence, a quasispecies structure, at a given time, may be summarized by aggregating the molecules (reads) that belong to each fitness type, in four fractions, defined as follows:

- Master: the fraction of molecules belonging to the most frequent haplotype; that is, the one present at the highest percentage;
- Emerging: the fraction of molecules present at a frequency >1% and less than the master percentage, belonging to haplotypes that are able to compete with the predominant one and possibly replace it;
- Low fitness: the fraction of molecules present at frequencies from 0.1% to 1%, belonging to haplotypes that have a low probability of progressing to higher frequencies;
- Very low fitness: the fraction of molecules present at frequencies <0.1% belonging to haplotypes with very low fitness and to defective genomes. The likely fate of these molecules individually is degradation, but the fraction is continuously fed with new very low fitness genomes produced by replication errors or by host editing activities.

QFF values were rarefied, as described above.

Distance between quasispecies

A quasispecies can be seen as a genetic population, and the methods used to study diversification in genetic populations can also be used to determine the distance or dissimilarity between different quasispecies. The method of Matoshi Nei[3] was used to compute the net genetic distance between quasispecies, D_A, from raw genetic distances between pairs of haplotypes in both quasispecies. The net distance, D_A, is the average distance between pairs of haplotypes or phenotypes in the two quasispecies, D_{XY}, corrected by the mean of intra-quasispecies genetic or phenotypic diversity of the two samples being compared.

The same method was used to compute the distance between quasispecies in terms of phenotypic distances, where the distance between pairs of phenotypes was computed by the method of Grishin[4] applied to BLOSUM80 matrix values. D_A values have been divided by the maximum in the series to obtain values in the range 0-1, and render comparable the evolution at the genetic and at the phenotypic level.

On the other hand, the similarity between pairs of sequential quasispecies was computed by comparing the haplotype/phenotype quasispecies distributions using the indices of common molecules (*Cm*), distribution overlap (*Ov*), and Yue-Clayton (*YC*)[5]. These similarities, normalized in the range 0-1, are transformed into dissimilarities by the rule: *Dissimilarity = 1 – similarity*.

From any of these distances or dissimilarities, quasispecies dendrograms and MDS maps are constructed to visualize the relationship between them.

Given that all haplotypes or phenotypes in all samples to be compared must be multiple aligned before computing distances between pairs of them, and the high number of haplotypes in the present study, an approximation was used. The top 50 (most abundant) haplotypes, or the top 20 phenotypes, of each sample were selected to be aligned and used in the computations. As the terms in the computation of distances are of order 2, their value quickly fade for very low frequencies, and the approximation is valid in the context of this study (see Figure 5). The impact of the approximation in terms of reads is also shown in Supplementary. Figure S4.

Fraction of synonymous reads and haplotypes

The values represented in Figure 3 have been rarefied to the minimum coverage of 147,463 reads, as described above.

Impact of amino acid changes in protein functionality

The observed amino acid mutations with respect to the master phenotype were evaluated in terms of probability of occurrence by the Fitch distance[6], which states the minimum number of nucleotide substitutions needed to cause the mutation; and in terms of functionality impact by the Grantham distance between amino acid pairs[7]. The Grantham distance between amino acids considers three factors: molecular volume, polarity, and molecular composition. Supplementary Figure S11 shows the combination of the two terms Fitch + Grantham, as a scale where to evaluate the putative impact of a mutation in protein functionality.

SUPPLEMENTARY RESULTS

Coverage and sample characteristics

Supplementary Table S1 summarizes clinical data and sequencing results of all samples in the study. We have studied between 147,463 reads of a single amplicon in ORF2 in sample S09, to 419,804 reads in sample S08. During RBV treatment, we observed a fluctuation in the number of haplotypes and in the frequency of the master sequence.

Master haplotypes vs master phenotypes

The first analysis concerns the confrontation of the dominant sequences at the nucleotide and at the amino acid level in each sequential sample. Figure 1 shows the UPGMA tree of the master haplotypes based on raw nucleotide distances in pairwise-deletion mode, and the corresponding tree with the master phenotypes based in distances computed from the BLOSUM-80 matrix.

At the nucleotide level, the same master haplotype was maintained until past 1162 days post-diagnosis (1162d, S07), although with declining frequencies. The sample at 1414d (S09) shows a different master at relatively low abundance (6.08%). The masters become different at each sequential sample since 1414d (S09). Notably the frequency increases to 59.67% at 1358d (S08), but decreases afterwards, remaining below 5%.

At the functional level (amino acid analysis), the same phenotype is observed until past 1162d (S07) at frequencies between 64.77% and 76.37%. At 1358d (S08) a different phenotype is observed at 75.05%. Finally, a new master phenotype is observed at 1414d (S09) and maintained till the last sample, but declining in frequency (62.3%, 41.0%, 35.4%) until the last sample in which it is expressed at higher frequency (66.9%).

The much larger frequencies of the master phenotypes compared to the master haplotypes, in the samples since 1414d (S09), suggest the presence of a set of haplotypes synonymous to the master phenotype.

As a complement, Supplementary Figure S2 shows the polymorphic sites in the comparison of master haplotypes and phenotypes; and Supplementary Figure S3 shows the track of masters, in frequency, in the sequential samples.

Quasispecies dendrograms

Aligning the top 50 haplotypes in each sample we obtain the matrix of pair-wise quasispecies genetic distances. Based on these distances a UPGMA quasispecies tree is constructed. On the other hand, by the alignment of the to 20 phenotypes in each sample we obtain the pair-wise quasispecies phenotype distances, and the corresponding UPGMA tree is constructed. Supplementary Figure S4 shows the two confronted dendrograms.

At the genetic level the sample at 1358d (S08) appears to be disruptive between the previous quasispecies and those that follow. The quasispecies in the last four samples are more closely related than expected from the master's UPGMA tree. At the functional level the UPGMA tree of the quasispecies phenotypes is similar in structure to the tree of the master's phenotypes, with larger distances between the last four samples, despite sharing a prevalent master phenotype.

Quasispecies fitness fractions and diversity

The QFF of the quasispecies at the genetic and phenotypic level are confronted in Figure 2, with data in Supplementary Table S2:

At the genetic level, the diversity is very high since the first sample, with most samples showing a fraction of reads for rare and very rare haplotypes above 50%, except for the sample at 1358d (S08). The fraction of reads for very rare haplotypes in the quasispecies increases at each sample, consistent with a mutagenic treatment, except for the sample at 1358d. Finally, the emerging fraction is taking substantial values in the last four samples.

At the functional level we see a different scenario, with master phenotypes above 50% in all samples, except for two. Of note the significant fraction of emerging phenotypes in the last four samples, showing the presence of functional phenotypes different to the master phenotype, which is shared by these samples.

As an alternative view to Figure 2, Supplementary Figure S5 shows the evolution in the master and emerging fractions, side by side, and Supplementary Figure S6 shows the number of rare haplotypes/phenotypes, side by side. The quasispecies diversity of each sample is evaluated in terms of the Hill numbers profile[2] and represented in Supplementary Figure S7 as a

complementary view of quasispecies structure and composition. The last four samples show the highest diversity levels at all q values.

Quasispecies population distance & quasispecies distribution similarity between sequential samples

The changes produced in a quasispecies after short periods of time may be followed by computing the similarity in the haplotype distribution between pairs of sequential samples. Three indices are computed: *Cm*, index of common molecules, *ov*, index of distribution overlap, and *yc*, index of Yue-Clayton[5]. These values are represented in Supplementary Figure S8.

At the genetic level the similarity between haplotype distributions is maintained high until the sample at 1358d (S08), with completely different quasispecies in the four samples that follow. On the other hand, at the functional level, the same differentiation is observed in the sample at 1358d (S08), but the next samples recover moderate values of similarity. These results are consistent with the changes in master sequences and frequencies shown in Figure 1.

The net genetic and phenotypic distances, D_A, between pairs of sequential samples are depicted in Supplementary Figure S9, showing a similar pattern of evolution between the two. The intra-quasispecies diversity of each sample is shown in Supplementary Figure S10, evidencing a big rise after 1358d.

Fraction of synonymous reads and haplotypes to the master phenotype

As commented above, the higher frequencies of the master phenotypes compared to the master haplotypes suggest the presence of multiple haplotypes synonymous to the master phenotype. In studying the fraction of synonymous reads to the master phenotype of each sample (Figure 3), we observe consistent values above 60%, except for two samples, 2065d (40.96%) and 2096d (35.43%). The last sample shows again a fraction above 60% (66.86%). On the other hand, the fraction of synonymous haplotypes to the master phenotype show values above 40% except for three samples; 1358d (30.90%), 2065d (34.35%) and 2096d (30.72%). The last samples increase to the highest value in the series at 57.34%. The ratio of number of haplotypes to number of phenotypes is given in Supplementary Table S3.

High multiplicity synonymous mutants

The relevancy of these synonymous haplotypes is further analysed by aggregating the reads of these synonymous haplotypes according to the number of substitutions with respect to the master haplotype in each sample (Supplementary Figure S4, Supplementary Table S4 and S5). All samples from 0d to 1162d show similar structure, although with increasing fractions at 1 and 2 substitutions. The sample at 1358d shows a prevalent master, followed by a significant fraction

at 1 substitution, and by tiny fractions with 2 to 7 substitutions. These fractions proliferate showing the 1414d sample with a small fraction of mater reads, and increased fractions with 2 to 6 substitutions. The proliferation of these high multiplicity mutants increases mainly in the 5 to 9 fractions in the next two samples. The atomization observed with the samples at 2065d and 2135d causes that the master haplotype expresses a phenotype different to the master phenotype since m0 is null. The last three samples in the series show significant fractions for 10 to 14 substitutions. They have been aggregated together in the m9.up fraction given the limited number of distinguishable colors.

Frequencies of top 10 haplotypes and phenotypes

Beyond the fraction of molecules expressing the master phenotype in each sample, which contribute to a higher frequency in the master phenotype with respect to the master haplotype, some samples show emerging phenotypes with enough functionality, given its frequency. These phenotypes are variants of the main phenotype where some amino acid has been changed by another resulting in a protein of similar chemical structure and functionality. This is particularly notorious in the last four samples (Figure 5).

Amino acid mutations

The probability to observe an amino acid mutation in a protein depends of two factors. The first, is the minimum number of nucleotide substitutions required to produce the mutation. The second is the chemical similitude between the two amino acids, in terms of molecular volume, polarity and chemical composition (Supplementary Figure S11). A high chemical similitude between the wild type amino acid and the new one, will likely not cause significant alterations in the 3D-chemical structure of the protein and in its functionality. In some cases, conservative substitutions may even enhance the function of a protein. For example, a conservative substitution in a binding site may improve the affinity of a protein for its ligand.

The mutations observed in master or emerging phenotypes (frequency above 1%) in the last four samples, with respect to the d0 master phenotype are shown in Supplementary Table S2. The most prevalent changes correspond to T → N and T → A, present in 27 and 23 different phenotypes, respectively. Observed aminoacidic changes require a single nucleotide substitution to occur (distance of Fitch=1). Most of the mutations correspond to a Grantham distance below Q1, five being below D1. These results suggest the conservative nature of these mutations.

Pattern of substitutions

The substitutions with respect to the consensus sequence were analysed in terms of its type and frequency, looking for the pattern of substitutions expected from the treatment with RBV. Most of the substitutions were transitions, as expected, but the substitutions C→T / C and G→ A / G

are only clearly prevalent in the last four samples (Supplementary Table S6). In samples where a master haplotype dominates the quasispecies, this analysis would be done computing the substitutions with respect to this master in the hope that most substitutions were produced from the master sequence. In the present scenario, the low frequency of the masters in most samples suggests that a better alternative would be the use of the consensus as reference. Nevertheless, the consensus is an artificial construction, and we may not exclude that its use could somehow blur the results.

REFERENCES

1. Gregori, J.; Soria, M.E.; Gallego, I.; Guerrero-Murillo, M.; Esteban, J.I.; Quer, J.; Perales, C.; Domingo, E. Rare Haplotype Load as Marker for Lethal Mutagenesis. *PLoS One* **2018**, *13*, doi:10.1371/journal.pone.0204877.
2. Gregori, J.; Colomer-Castell, S.; Campos, C.; Ibañez-Lligoña, M.; Garcia-Cehic, D.; Rando-Segura, A.; Adombie, C.M.; Pintó, R.; Guix, S.; Bosch, A.; et al. Quasispecies Fitness Partition to Characterize the Molecular Status of a Viral Population. Negative Effect of Early Ribavirin Discontinuation in a Chronically Infected HEV Patient. *Int J Mol Sci* **2022**, *23*, 14654, doi:10.3390/ijms232314654.
3. Nei, M. *Molecular Evolutionary Genetics*; Columbia University Press: New York, 1987; ISBN 9780231886710.
4. Grishin, V.N.; Grishin, N. V Euclidian Space and Grouping of Biological Objects. *Bioinformatics* **2002**, *18*, 1523–1534, doi:10.1093/bioinformatics/18.11.1523.
5. Gregori, J.; Ibañez-Lligoña, M.; Quer, J. Quantifying In-Host Quasispecies Evolution. *Int J Mol Sci* **2023**, *24*, doi:10.3390/IJMS24021301.
6. Fitch, W.M. An Improved Method of Testing for Evolutionary Homology. *J Mol Biol* **1966**, *16*, 9–16, doi:10.1016/s0022-2836(66)80258-9.
7. Grantham, R. Amino Acid Difference Formula to Help Explain Protein Evolution. *Science* **1974**, *185*, 862–864, doi:10.1126/science.185.4154.862.

SUPPLEMENTARY FIGURES

Supplementary Figure S1: Time line of interventions, samplings and viral loads, in days since first evidence. Only viral loads corresponding to samples are shown. The first EOT shows negativization of HEV RNA, but is followed by a relapse (RLP) after few weeks.

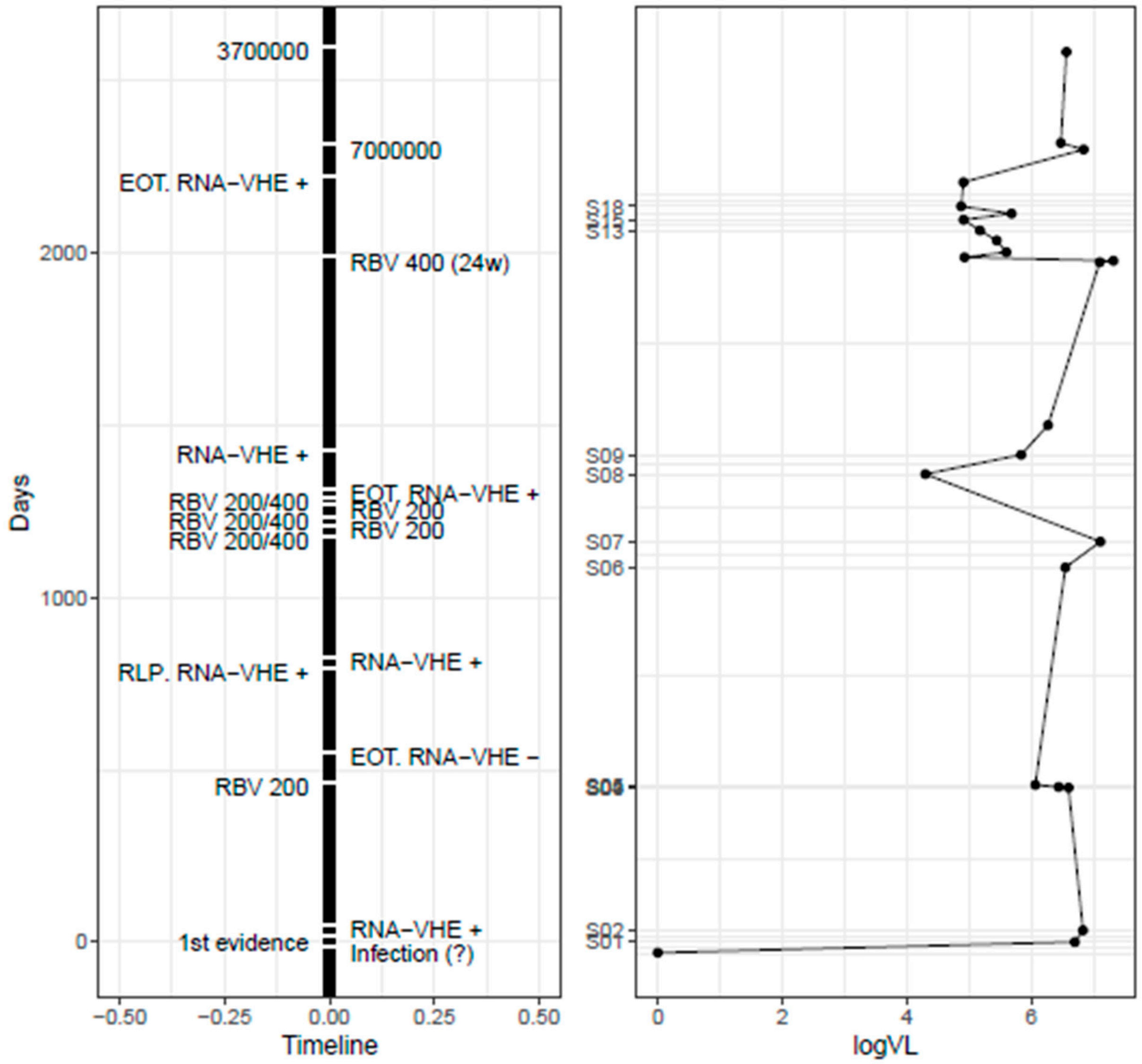

Supplementary Figure S2: Polymorphic sites in the comparison of A) master haplotypes and B) phenotypes. Positions within the amplicon are given in abscissa (nt 6347-6709, aa 409-529).

A

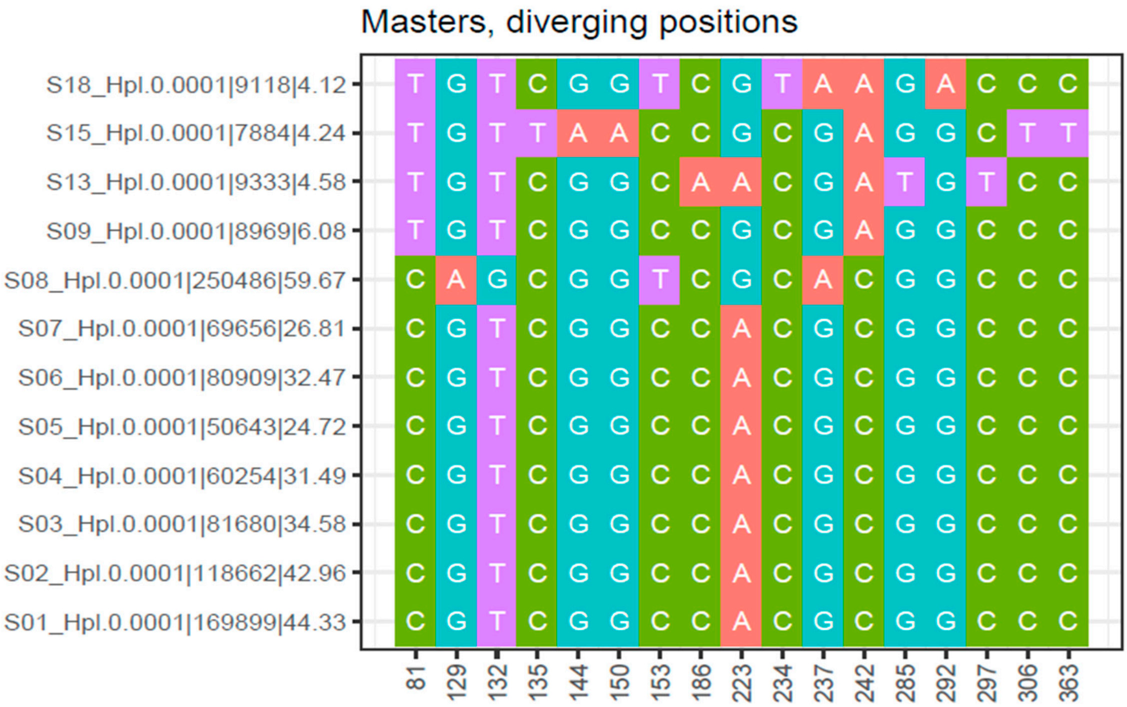

B

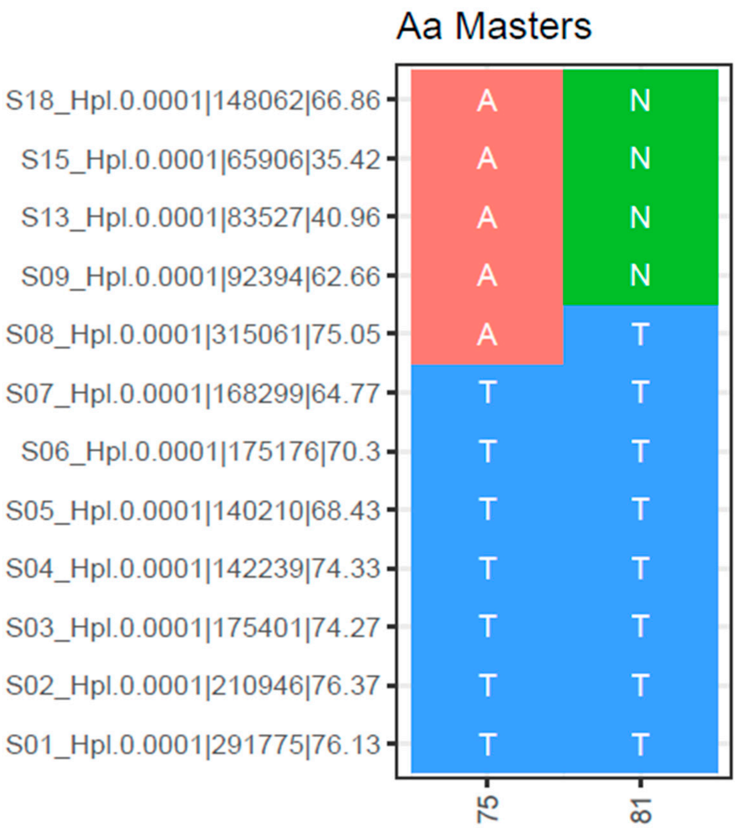

Supplementary Figure S3: Track in frequencies of the master haplotypes in samples evolution.

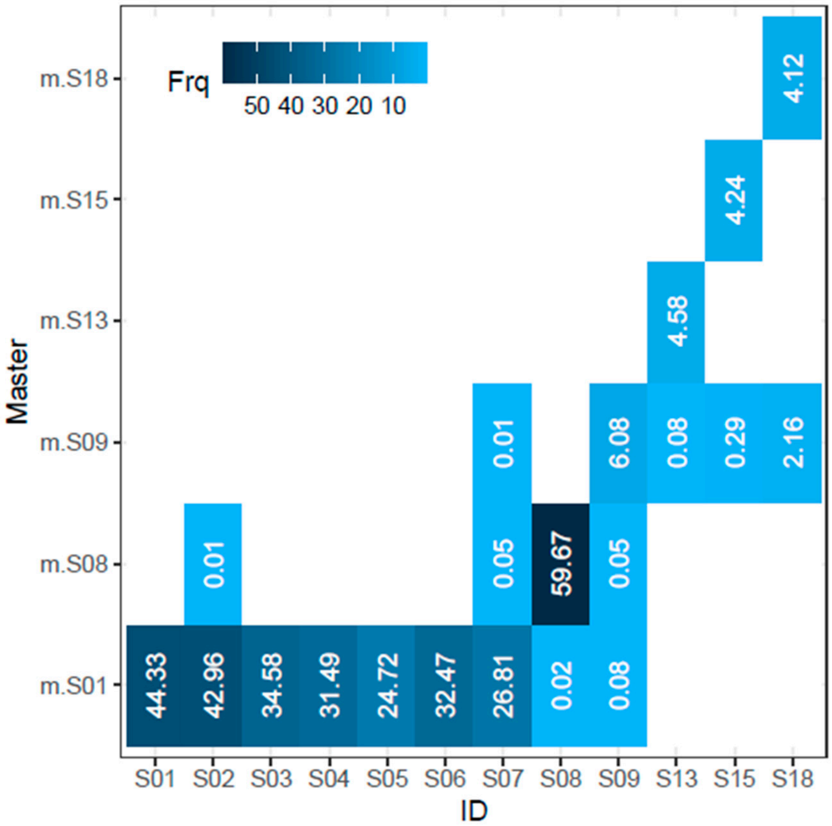

Supplementary Figure S4: (A) UPMA tree with the quasispecies represented by the top 50 haplotypes of all samples. (B) Corresponding tree with the quasispecies represented by the top 20 phenotypes of all samples. The percentages besides the sample labels give the fraction of reads in the quasispecies corresponding to the top 50 haplotypes, or top 20 phenotypes, which were multiple aligned to compute the quasispecies distances.

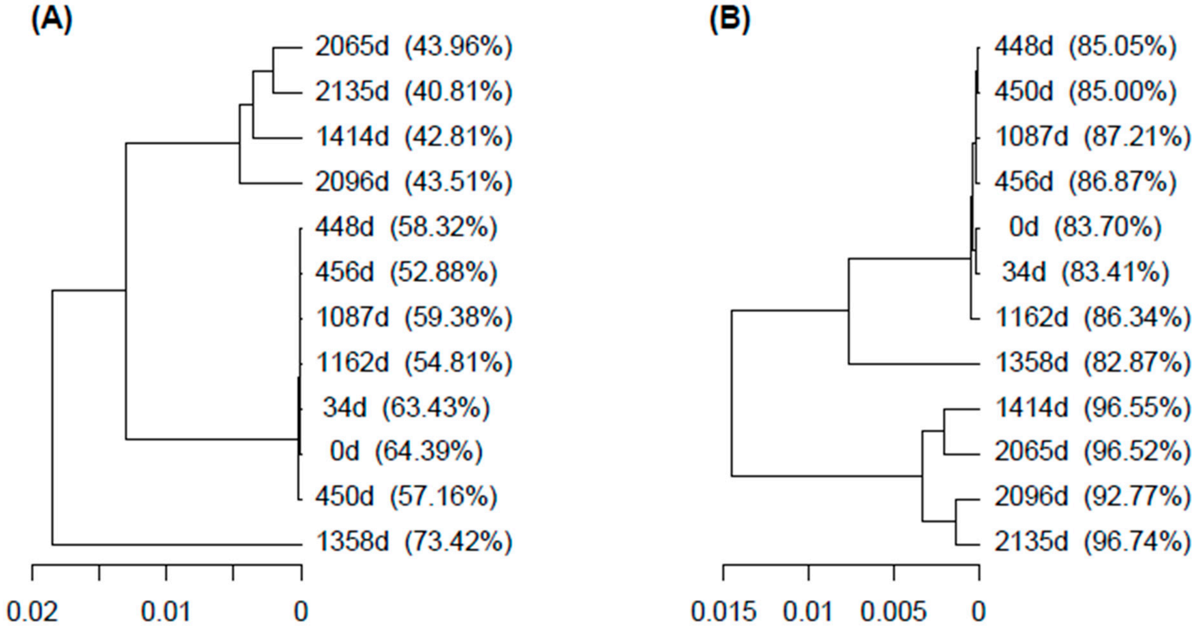

Supplementary Figure S5: Changes in master and emerging fractions. Genetic and phenotypic levels.

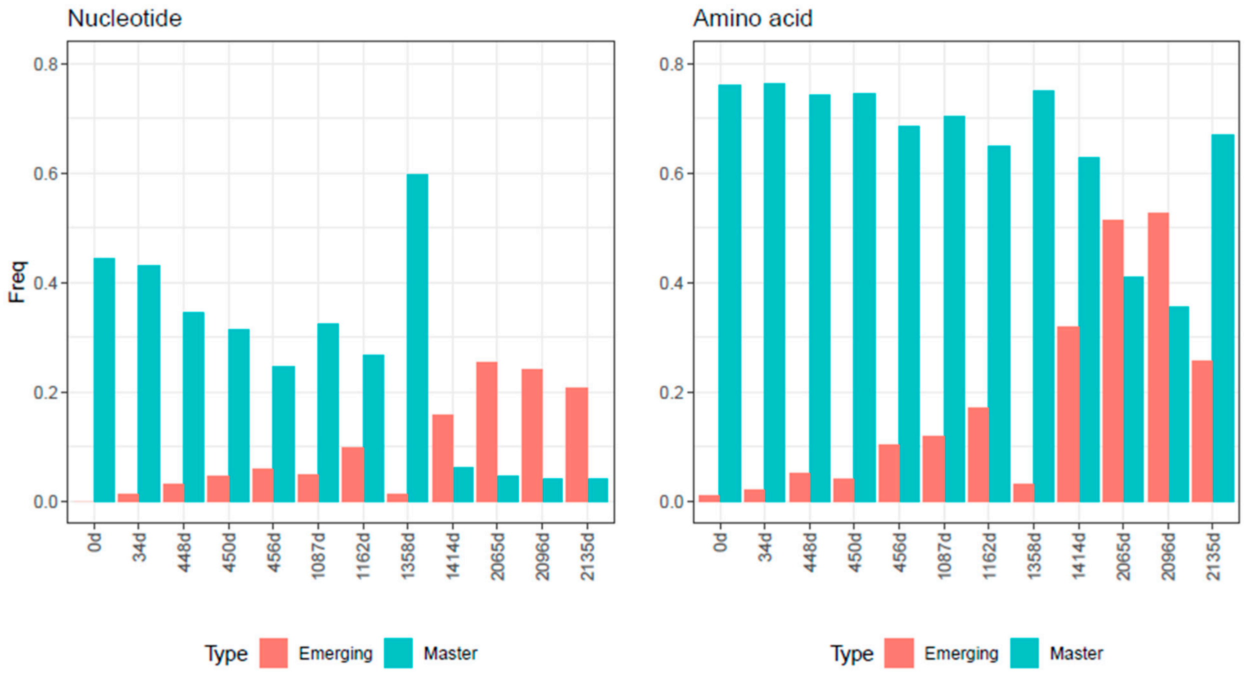

Supplementary Figure S6: Evolution in the number of rare haplotypes. Genetic and phenotypic levels.

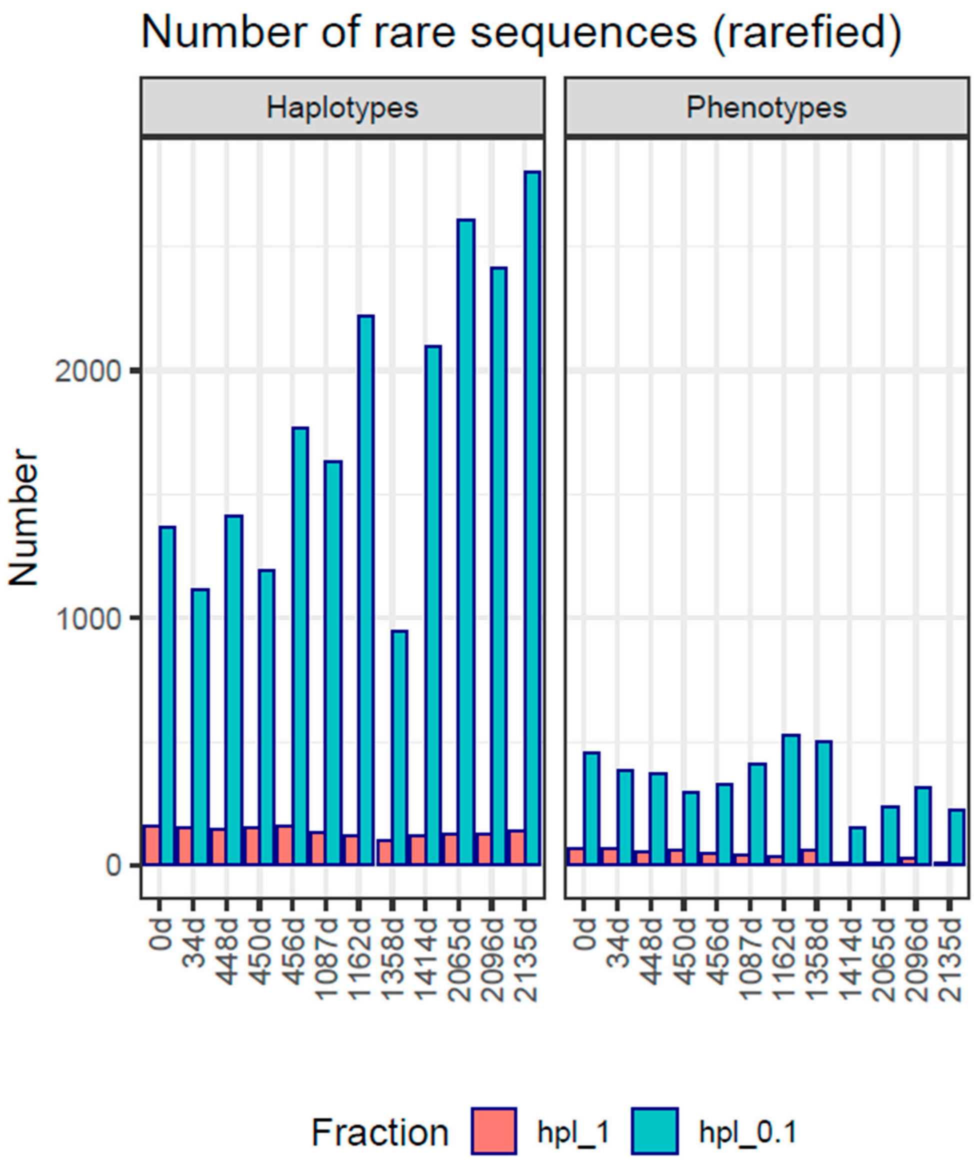

Supplementary Figure S7: Quasispecies diversity as Hill numbers profiles. Only values at q = 0, 1, 2, and Infinity are represented.

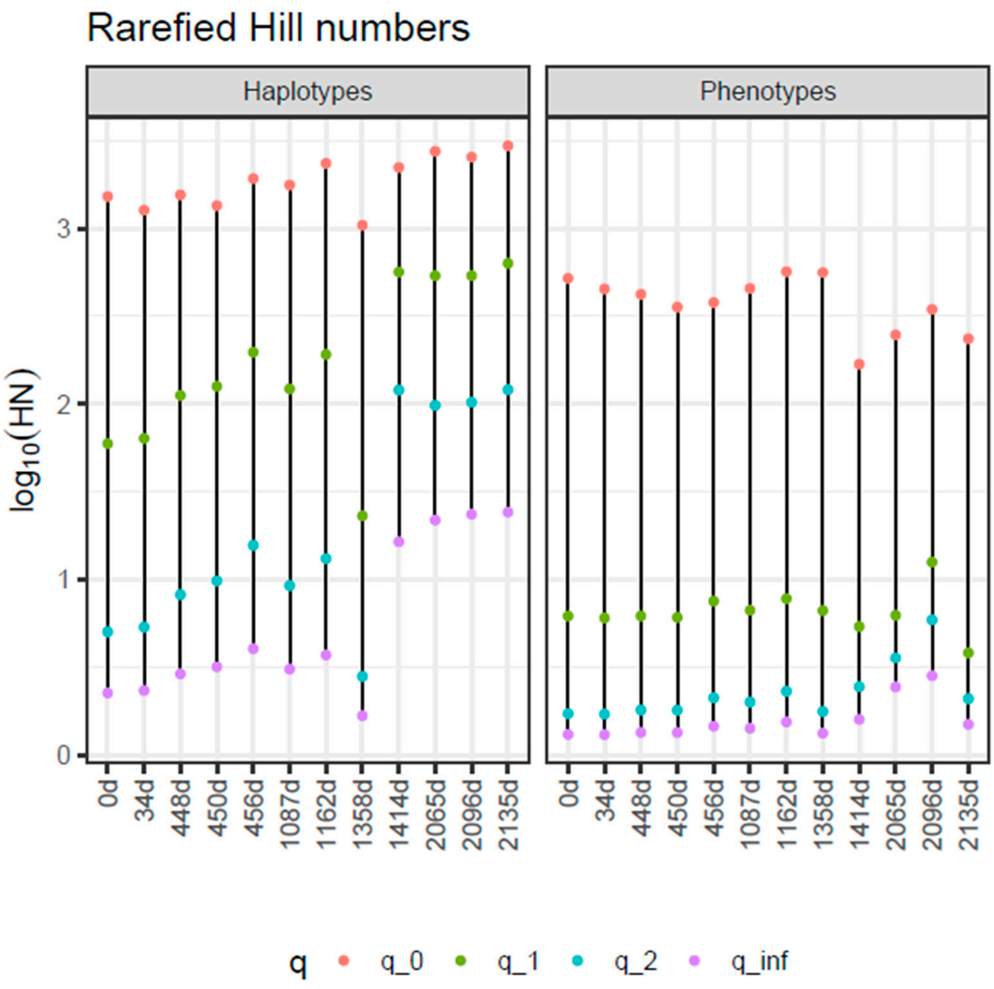

Supplementary Figure S8: Similarities in haplotype or phenotype quasispecies distribution between sequential samples. The 50 top haplotypes, and the 20 top phenotypes are considered. Cm: index of common molecules, ov: index of overlap, yc: index of Yue-Clayton. Similarity values are represented in the mid point between samples.

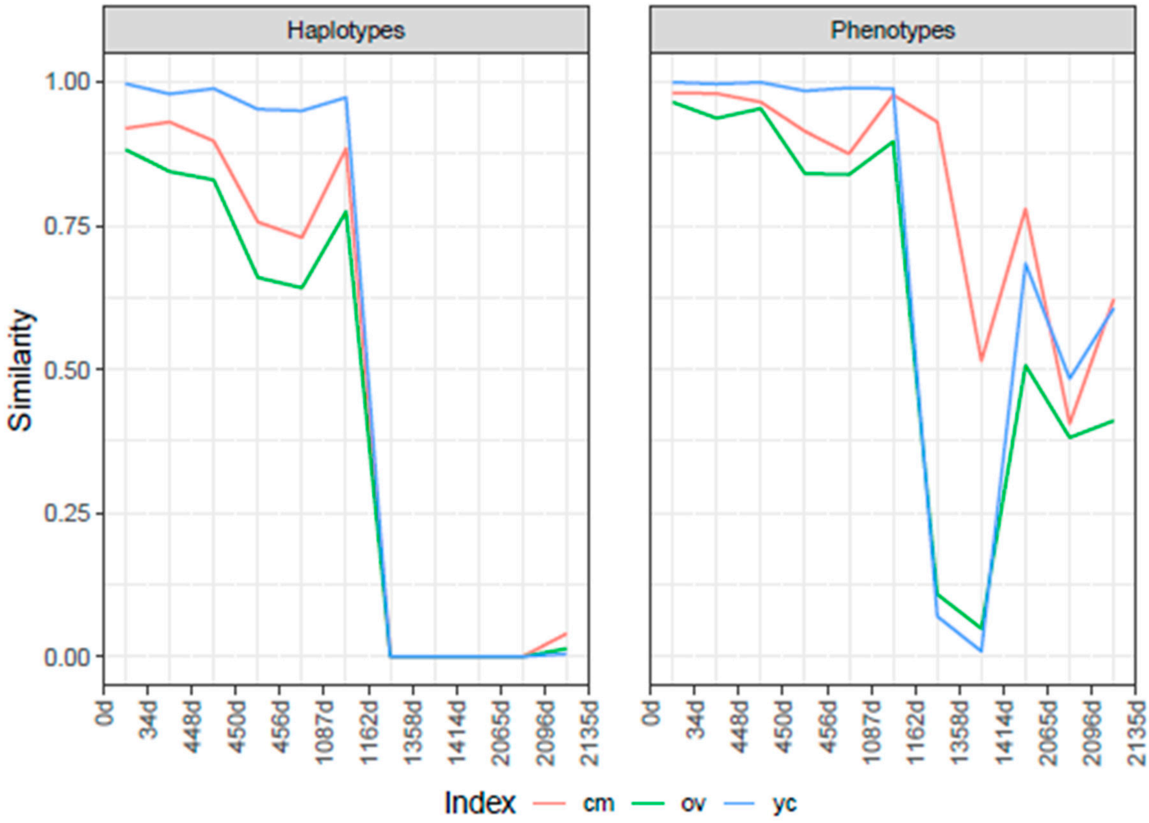

Supplementary Figure S9: Net genetic and phenotypic distance (D_A), between pairs of sequential samples. The 50 top haplotypes, and the 20 top phenotypes are considered. D_A values have been scaled to the maximum value in the sequence to render comparable genetic and phenotypic distances.

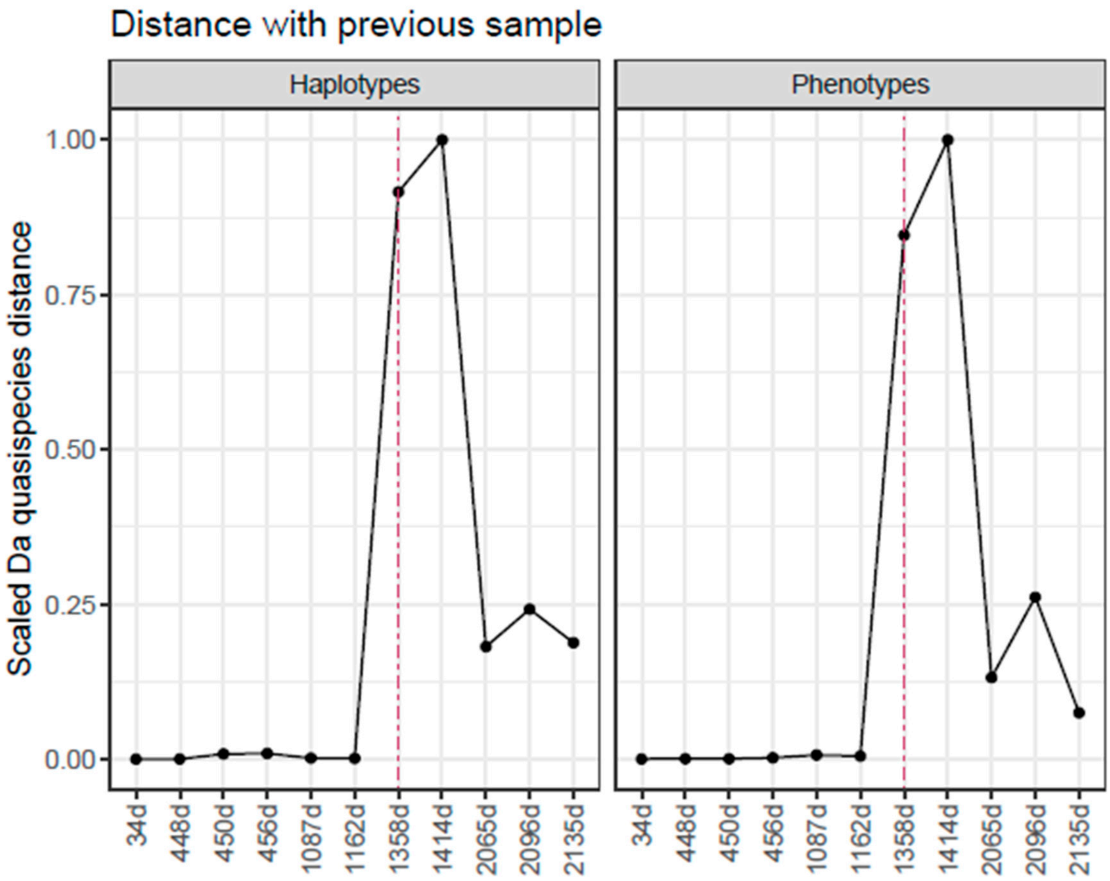

Supplementary Figure S10: Nucleotide and amino acid diversity of each sample. Values are scaled to the maximum value in the series to render comparable genetic and phenotypic values.

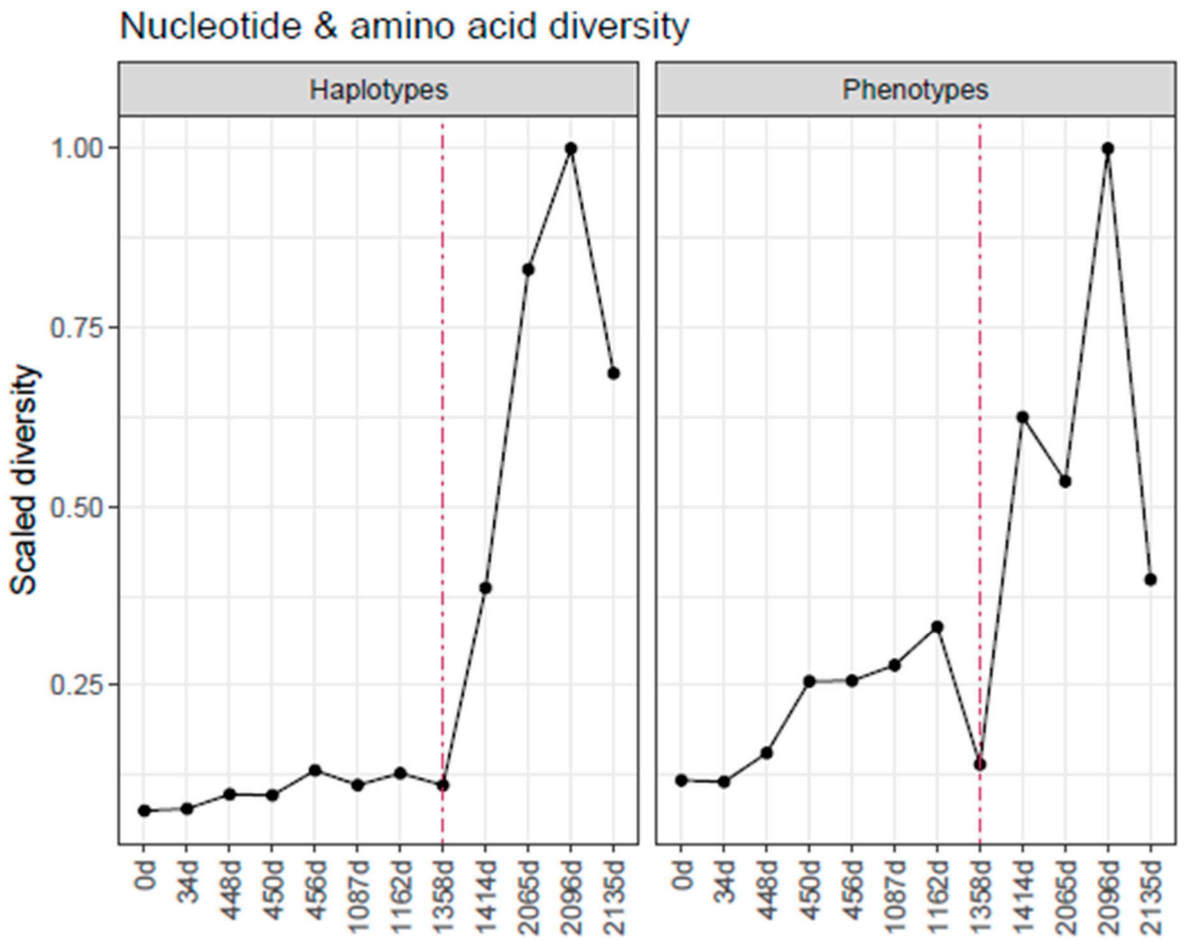

Supplementary Figure S11: Table of amino acid mutations sorted first by the Fitch distance (gray background) and then by the Grantham distance (blue shade), in increasing order of values. The mutations in Table 2 with N>=2 have been marked with a circle. The mutations nearest to the left, in each row, represent those expected to occur with higher chance and less impact on protein function. The lighter the blue color of the amino acid letter, the smaller the expected impact on protein functionality.

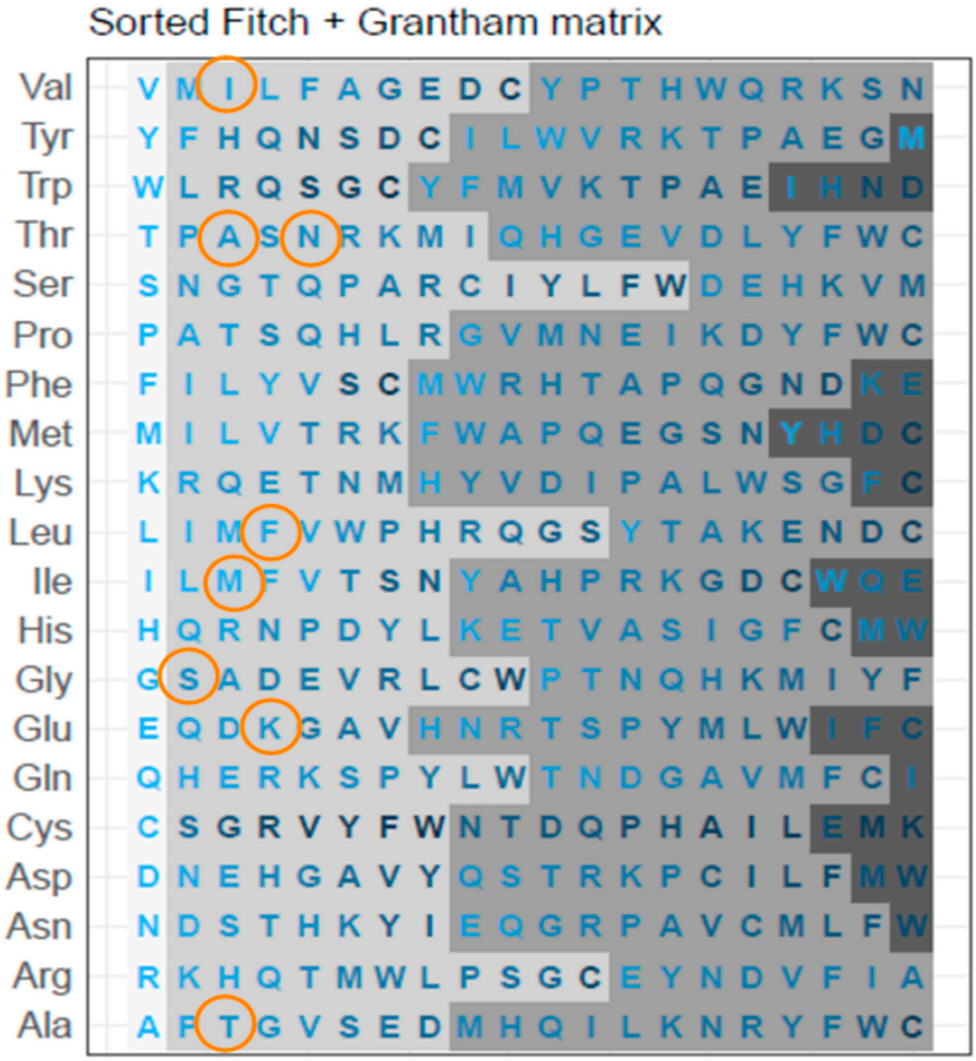

SUPPLEMENTARY TABLES

Supplementary Table S1: Days: days since first evidence, iWks: Interval in weeks between samples; Label: sample label used in figures and table; RBV: regimen of treatment; logVL base 10 logarithm of viral load; nReads: total number of reads in sample; nHpl: number of haplotypes in quasispecies; RdMaster: reads for the master haplotype in each sample.

ID	Date	Days	iWks	Label	RBV	logVL	nReads	nHpl	RdMaster
S01	13 November 2012	0	0	0d	-	6.70	383,263	1,526	169,899
S02	17 December 2012	34	4.9	34d	-	6.84	276,199	1,271	118,662
S03	4 February 2014	448	59.1	448d	200	6.60	236,181	1,554	81,680
S04	6 February 2014	450	0.3	450d	200	6.44	191,367	1,350	60,254
S05	12 February 2014	456	0.9	456d	200	6.07	204,883	1,927	50,643
S06	5 November 2015	1,087	90.1	1087d	-	6.55	249,191	1,768	80,909
S07	19 January 2016	1,162	10.7	1162d	200/400	7.11	259,841	2,348	69,656
S08	2 August 2016	1,358	28.0	1358d	-	4.30	419,804	1,047	250,486
S09	27 September 2016	1,414	8.0	1414d	-	5.84	147,463	2,227	8,969
S13	10 July 2018	2,065	93.0	2065d	400	5.18	203,926	2,751	9,333
S15	10 August 2018	2,096	4.4	2096d	400	4.91	186,055	2,549	7,884
S18	18 September 2018	2,135	5.6	2135d	400	4.88	221,446	2,956	9,118

Supplementary Table S2: Rarefied quasispecies fitness fractions at the genetic and phenotypic levels

	Haplotypes				*Phenotypes*			
ID	**Master**	**Emerging**	**RHL_1_0.1**	**RHL_0.1**	**Master**	**Emerging**	**RHL_1_0.1**	**RHL_0.1**
0d	0.4433	0.0000	0.3652	0.1915	0.7613	0.0113	0.1263	0.1012
34d	0.4297	0.0129	0.3588	0.1987	0.7637	0.0203	0.1093	0.1066
448d	0.3458	0.0300	0.3659	0.2583	0.7427	0.0519	0.1006	0.1048
450d	0.3149	0.0438	0.3846	0.2567	0.7433	0.0407	0.1233	0.0927
456d	0.2471	0.0597	0.4317	0.2614	0.6843	0.1037	0.1353	0.0768
1087d	0.3247	0.0538	0.3636	0.2580	0.7029	0.1191	0.0840	0.0940
1162d	0.2680	0.1023	0.2967	0.3330	0.6477	0.1715	0.0667	0.1141
1358d	0.5967	0.0118	0.1846	0.2069	0.7505	0.0299	0.0990	0.1206
1414d	0.0608	0.1589	0.3429	0.4375	0.6266	0.3179	0.0200	0.0355
2065d	0.0458	0.2536	0.2675	0.4331	0.4096	0.5140	0.0381	0.0383
2096d	0.0424	0.2461	0.2701	0.4414	0.3542	0.5268	0.0747	0.0442
2135d	0.0412	0.2061	0.3101	0.4427	0.6686	0.2565	0.0378	0.0370

Supplementary Table S3: Number of haplotypes, phenotypes and the ratio between them, showing the level of synonymous haplotypes in each quasispecies.

ID	*Haplotypes*	*Phenotypes*	*Ratio*
0d	1526	521	2,93
34d	1271	453	2,81
448d	1554	422	3,68
450d	1350	357	3,78
456d	1927	379	5,08
1087d	1768	456	3,88
1162d	2348	568	4,13
1358d	1047	562	1,86
1414d	2227	169	13,18
2065d	2751	248	11,09
2096d	2549	346	7,37
2135d	2956	236	12,53

Supplementary Table S4: Number of haplotypes at increasing number of substitutions with respect to the master haplotype in each quasispecies. Only haplotypes synonymous to the master phenotype are considered.

nHpl	***Substitutions vs master haplotype in synonymous vs master phenotype***														
	**0**	**1**	**2**	**3**	**4**	**5**	**6**	**7**	**8**	**9**	**10**	**11**	**12**	**13**	**14**
0d	1	211	465	8	0	0	0	0	0	0	0	0	0	0	0
34d	1	202	377	9	0	0	0	0	0	0	0	0	0	0	0
448d	1	190	547	25	4	0	0	0	0	0	0	0	0	0	0
450d	1	160	521	22	3	0	0	0	0	0	0	0	0	0	0
456d	1	141	521	89	3	2	0	0	0	0	0	0	0	0	0
1087d	1	157	521	24	3	2	0	0	0	0	0	0	0	0	0
1162d	1	185	521	35	2	0	0	0	0	0	0	0	0	0	0
1358d	1	221	521	15	12	9	7	9	0	0	0	0	0	0	0
1414d	1	86	521	372	186	87	29	6	1	0	0	0	0	0	0
1500d	1	97	521	279	103	8	0	0	0	0	0	0	0	0	0
1973d	1	48	521	128	80	12	6	3	2	0	0	0	0	0	0
2065d	0	1	521	10	29	71	119	233	170	150	77	54	19	6	2
2096d	1	48	521	45	67	70	79	109	120	92	70	26	12	5	2
2114d	1	34	521	190	139	106	66	28	17	4	2	0	0	0	0
2135d	0	1	521	60	99	152	319	404	305	175	83	37	20	13	5

Supplementary Table S5: Mutation load. Reads: number of reads in sample; Seqs: Number of unique sequences, genotype and phenotype level. Subst: Total number of substitutions with respect to the master haplotype. SubsLoad: ratio Subst/Seqs; Muts: total number of mutations observed with respect to the master phenotype; MutLoad: ratio Muts/Seqs.

		Haplotypes			***Phenotypes***		
ID	**Reads**	**Seqs**	**Subst**	**SubsLoad**	**Seqs**	**Muts**	**MutLoad**
0d	383.263	1.526	237.021	0,618	521	206.459	0,539
34d	276.199	1.271	173.309	0,627	453	144.478	0,523
448d	236.181	1.554	188.063	0,796	422	110.619	0,468
450d	191.367	1.350	171.584	0,897	357	121.594	0,635
456d	204.883	1.927	219.934	1,073	379	107.134	0,523
1087d	249.191	1.768	221.522	0,889	456	154.246	0,619
1162d	259.841	2.348	260.635	1,003	568	142.748	0,549
1358d	419.804	1.047	305.766	0,728	562	296.364	0,706
1414d	147.463	2.227	394.977	2,678	169	80.240	0,544
2065d	203.926	2.751	1.283.855	6,296	248	163.983	0,804
2096d	186.055	2.549	1.512.102	8,127	346	184.330	0,991
2135d	221.446	2.956	1.431.794	6,466	236	90.838	0,410

Supplementary Table S6: Number of substitutions by type, with respect to consensus sequence in each quasispecies.

	Substitution freq.				*Wild type freq.*			
	**C->T**	**G->A**	**T->C**	**A->G**	**C**	**G**	**T**	**A**
0d	471	176	1078	292	103	83	98	79
34d	418	181	752	294	103	83	98	79
448d	593	219	1148	449	103	83	98	79
450d	646	214	905	405	103	83	98	79
456d	1135	647	1349	421	103	83	98	79
1087d	886	467	982	474	103	83	98	79
1162d	1108	785	1151	670	103	83	98	79
1358d	353	437	344	331	102	83	98	80
1414d	3115	2877	679	240	102	84	98	79
2065d	4912	5781	1104	537	101	84	99	79
2096d	6607	6194	441	360	101	84	99	79
2135d	6102	5541	587	382	101	84	99	79
